# Chronic IVC occlusion caused by unopened filter after conversion: case report and literature review

**DOI:** 10.3389/fcvm.2024.1520836

**Published:** 2025-01-30

**Authors:** Shi Sheng, Yiqing Li

**Affiliations:** Department of Vascular Surgery, Union Hospital, Tongji Medical College, Huazhong University of Science and Technology, Wuhan, China

**Keywords:** inferion vena cava, deep venous thrombolism, VenaTech convertible filter, pulmonary embolism, percutaneous mechanic thrombectomy

## Abstract

**Background:**

The VenaTech Convertible Vena Cava Filter (VTCF) is a device designed for insertion into the inferior vena cava (IVC) to prevent life-threatening pulmonary embolism (PE). Upon removal of its retrieval hook, the filter's legs are intended to expand, forming a stent-like structure that is suitable for long-term residence in the human body. However, in clinical practice, the filtering legs do not always expand fully, and the long-term effects on patients remain insufficiently studied.

**Materials and methods:**

This report presents the case of a male patient with thrombophilia, in whom the VTCF failed to expand completely after conversion, resulting in IVC occlusion and the development of acute deep vein thrombosis (DVT) in the lower limbs. A review of the relevant literature is also provided.

**Conclusion:**

The inability of the filtering legs to fully expand after retrieval hook removal highlights a design limitation of the VTCF, necessitating proactive management during conversion to ensure complete expansion. For younger or thrombophilic patients, careful evaluation of the filter's suitability and extended follow-up are crucial to optimize outcomes.

## Background

PE significantly impacts human health, causing approximately 300,000 deaths annually in the United States alone (1). IVC filters are widely used in clinical settings as an effective method for intercepting dislodged clots from the lower limbs to prevent acute massive PE. After controlling high-risk factors for thrombosis, the filter is generally considered for removal to reduce complications associated with long-term placement.

Based on the VenaTech LP (B. Braun) permanent IVC ﬁlter design, the VTCF consists of eight stabilizing legs, eight ﬁltering legs. The core design of the VTCF features a detachable end at the filter head. Once the retrieval hook is removed, the filtering legs are theoretically intended to gradually unfold, eventually forming a stent-like structure that adheres to the IVC wall, thereby minimizing the impact on blood flow. However, after the retrieval hook is removed, the filtering legs do not always return to their fully open state for various reasons. This may potentially affect the hemodynamics of the IVC.

This case is the first publicly reported instance where, following a successful filter conversion, the failure of the filtering legs to fully expand led to IVC occlusion, accompanied by the formation of acute deep vein thrombosis in the lower extremities six years later.

## Case report

**Figure 1:**
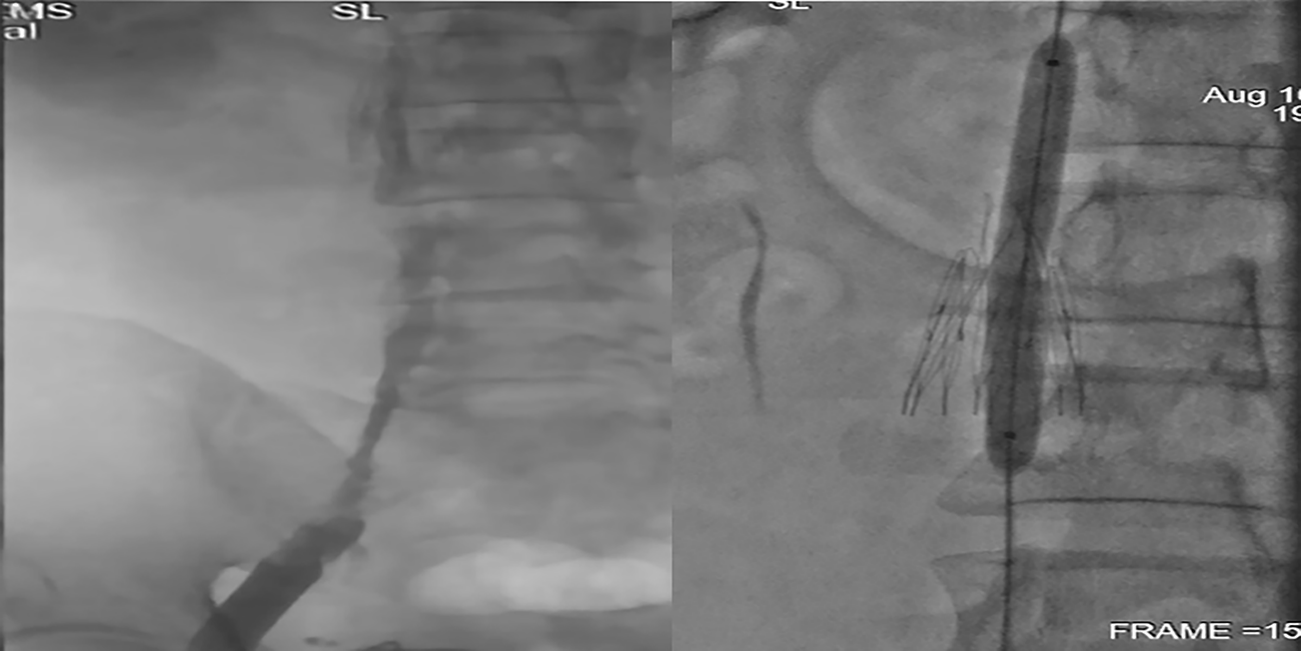
(left: CTV showed the filtering legs are still unopened while the hook were moved 6 years ago; right: DSA showed the same with CTV).

A 34-year-old male patient was admitted to our vascular surgery department with complaints of discomfort and swelling in his right lower limb, which had persisted for three days. Physical examination revealed mild swelling in the right lower limb, accompanied by increased skin temperature. Laboratory tests showed a D-dimer level of 1.48 mg/L. Ultrasound imaging revealed uneven filling of the IVC and bilateral iliac veins, along with fresh thrombosis in the right femoral vein and chronic thrombosis in the left common femoral vein, which was partially recanalized. The Computed Tomography Pulmonary Angiography (CTPA) did not detect any pulmonary artery thrombosis. However, a computed tomography (CT) scan of the IVC showed that the retrieval hook of a previously placed IVC filter had been removed, though the filter's legs had not expanded (Figure 1).

Further medical history revealed that six years prior, the patient had an IVC filter implanted due to DVT, and genetic testing confirmed a diagnosis of thrombophilia (gene: PROS1; mutation site: Unknown_000313:c.-190C>G). Four months after the filter insertion, it was “successfully” converted at another hospital. In 2017, the patient was admitted to our hospital with hematuria and was diagnosed with membranous nephropathy through renal biopsy. The patient has not been on long-term anticoagulant or corticosteroid therapy.

Given the patient's significant thrombotic burden, and with the consent of the patient, we performed a percutaneous mechanical thrombectomy (PMT) on the third day of admission. The aim was to remove as much thrombus as possible and to expand the filter's legs, if feasible. Since the patient did not exhibit symptoms in the left leg, PMT was not planned for the left iliac and femoral veins.

**Figure 2:**
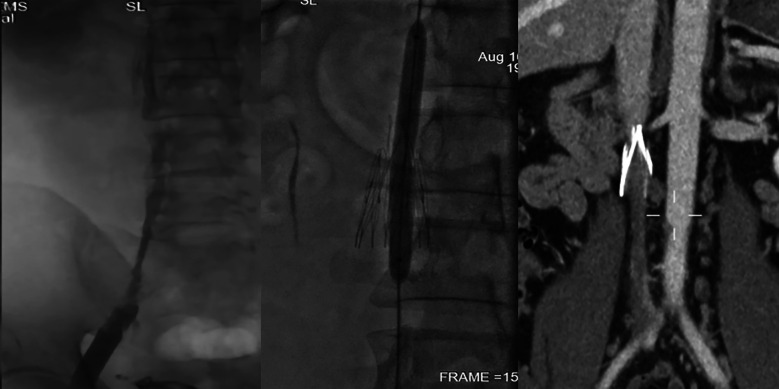
(left: 12*80 mm balloon dilated; middle: IVC partly revascularized; right: IVC partly occluded again 3 months post-surgery).

During the procedure, access was gained through the right popliteal vein. A Zelante catheter equipped with an AngioJet thrombectomy system (Boston Scientific) was used to repeatedly suction thrombi from the IVC and right iliac and femoral veins. Balloon dilation of the inferior vena cava (IVC) lumen was then performed using a series of balloons (3 × 150 mm, 8 × 80 mm, and 12 × 80 mm, Boston Scientific), followed by the administration of 100,000 units of urokinase directly into the lesion area. After 20 min of thrombolysis, a second round of percutaneous mechanical thrombectomy (PMT) was conducted. The procedure successfully reopened the right femoral vein and the right external iliac vein. However, only partial recanalization was achieved in the IVC and the right common iliac vein (Figure 2).

Given the likely chronic occlusion of the IVC and bilateral iliac veins, simple thrombus aspiration was insufficient to achieve optimal results. Further lumen recanalization would only be possible with stent placement. However, due to the patient's history of thrombophilia, age, and refusal of stent placement, we ultimately decided against its use. During the procedure, we attempted to expand the filter's legs using various balloons and pigtail catheter, but these efforts were unsuccessful. Considering that the acquired narrow lumen would soon become occluded without a stent and that fully opening the filter's legs would not alter this outcome, we decided to abandon further attempts via internal jugular vein access to avoid unnecessary iatrogenic injury

The patient was maintained on anticoagulation therapy during hospitalization and advised to continue oral anticoagulants long-term after discharge. Three months post-surgery, the patient underwent follow-up examination. As expected, the IVC remained filled with chronic thrombosis, but limb swelling had significantly improved. (Figure 2) If severe symptoms recur despite strict anticoagulation, stent deployment may be considered as an option (Figure 2).

## Discussion and review

DVT is a common clinical condition, and PE resulting from dislodged DVT is the third leading cause of cardiovascular death, following myocardial infarction and stroke (2). To prevent fatal PE, the use of filters has become increasingly widespread. However, once the high-risk period for PE has passed, the continued presence of a filter in the body may lead to various complications. The 2005 PREPIC study first suggested that, although permanent filter placement significantly reduces the incidence of PE, it also increases the incidence of lower limb DVT (3). Subsequent studies have shown that long-term filter placement can cause damage to the IVC wall, surrounding organs, filter tilting, filter fractures, and even PE, etc. (4, 5).

In response to these concerns, the concept of “convertible filters” was introduced in the 2006 Society of Interventional Radiology treatment guidelines. Convertible filters are defined as “permanent filters that can be structurally altered after implantation to no longer function as filters” (6). Based on this concept, B. Braun initiated research and development, and in 2011 (7), animal studies demonstrated the feasibility of converting a filter into a device that does not interfere with blood flow. This led to the introduction of the VTCF in 2016, developed from the permanent VTLP filter.

The VTCF features a unique design: its filtering legs are fixed within a removable locking device at the top, where the retrieval hook is located. This design allows the retrieval hook to be independently removed, releasing the filtering legs to expand naturally into a stent-like configuration and remain in the IVC. The advantage of this design is clear. In contrast, other retrievable filters, when removed, typically require peeling the filtering legs from the IVC wall. This “peeling” process becomes more difficult with prolonged filter placement due to intimal hyperplasia and adhesions. In some cases, this can result in significant tension on the IVC, leading to arrhythmias, lumen rupture, filter disintegration, or even dislodgement, causing iatrogenic PE and other serious adverse events (4, 5). Many long-term filters cannot be removed smoothly due to these complications, with literature reporting a failure rate as high as 43% (8). The VTCF design successfully avoids this issue by shifting the “peeling” process from the IVC wall and stabilizing legs to between the retrieval hook and filtering legs, thus minimizing damage to the IVC wall. This feature enables longer-term filter conversion, allowing it to function as a thrombus filter while offering an extended retrieval window and a safer retrieval process. It may be useful in patients with an indeterminate duration of VTE risk.

Although the VTCF theoretically offers significant design advantages, its use in clinical settings is less widespread compared to retrievable filters, and relevant studies are limited and often lack rigor. With the exception of one multicenter prospective single-arm study, most are single-center retrospective investigations. The published research indicates VTCF conversion success rates ranging from 92.7% to 100% (9–14), which is notably higher than the success rates of 76% reported for other types of retrievable filters in recent literature (15).

The studies reviewed in this research suggest that the filtering legs do not automatically open fully after the retrieval hook is removed, with the specific proportions varying widely across studies (ranging from 0% to 37%) (9–14) (see Table 1). Some literature does not explicitly address this phenomenon, merely describing it as a “successful conversion.” Due to the lack of a precise definition of successful conversion, some cases that are considered successful conversions may simply involve the retrieval of the hook, without fully expanding the filtering legs. As a result, the angle between the filtering legs and the vessel wall may remain unchanged after the conversion.

**Table 1:** Relevant data of VTCF conversion from published papers.

Author	All subjects	Subjects with conversion attempt	Days to conversion	Successful conversion rate	Completely-open rate	Follow-up time (month)	Users of accessory techniques
Dai, Li et al. (9)	31	30	11–21	100% (30/30)	100% (30/30)	5–17	3.3% (1/30)
Li, Dou et al. (10)	115	23	4–155	95.7% (22/23)	95.4% (21/22)	1–6	87% (20/22)
Ke, Huang (11)	103	27	5–145	96.3% (26/27)	63% (17/26)	6–12	Unknown
Shan, Chen et al. (12)	31	31	60	100% (31/31)	Unknown	6–22	Unknown
Dou, Zheng et al. (13)	52	48	>60	100% (48/48)	100% (48/48)	Unknown	81.25% (39/48)
Lin, Hom et al. (14)	149	96	15–391	96.9% (93/96)	95.7% (89/93)	6	82.3% (79/96)

This issue was initially observed in animal studies, where 60% of cases could not naturally open fully without balloon assistance and required further surgical intervention (7). In clinical practice, similar maneuvers often involve additional techniques, such as using the curvature and stiffness of catheters (e.g., pigtail or cobra catheters) to rotate, stir, and gently pull to separate the adhered filtering legs. Alternatively, an appropriate balloon may be used to expand the filtering legs by slowly pulling upward. Despite these techniques, a significant proportion (4.3%–37%) of patients still fail to achieve full expansion of the filtering legs (11, 14). Given that some literature does not report the status of the filtering legs’ expansion, the actual percentage may be higher.

The exact causes of this phenomenon are not yet fully understood. Some studies believe that there may be two possible reasons: first, adhesion and encapsulation caused by chronic thrombus and local intima hyperplasia around the filtering legs; second, the angle between the stabilizing legs and the filter legs, which persists due to endothelization on the surface of the stabilizing legs (11). Animal experiments have shown that within four weeks, the areas of the filtering legs in contact with the IVC wall become completely encapsulated by proliferative tissue, maintaining a persistent angle with the IVC wall (7). Further research is needed to clarify the underlying mechanisms.

Regardless of the cause, filtering legs that fail to fully expand negatively impact blood flow in the IVC. Computational fluid dynamics studies (16) simulating the effects of various filter states on blood flow have shown that fully expanded filtering legs produce flow conditions similar to those seen when no filter is present. In contrast, if the filtering legs does not fully expand, blood flow may be adversely affected by the filtering legs. This effect is positively correlated with the angle between the filtration feet and the wall of the IVC. Even if at very small angles to the vein wall, they continue to significantly impede blood flow, leading to platelet aggregation, thrombosis formation, and other complications (17).

In previous literature, cases of IVC thrombosis have been reported only among patients whose filters were either unsuccessfully converted or not converted at all. Unfortunately, the limited studies on VTCF, whether multicenter or single-center, typically have follow-up periods of only about six months, with the longest being just 22 months. Therefore, the long-term impact of incompletely opened filtering legs on hemodynamics remains unknown. In this case, the patient successfully underwent filter conversion six years ago but later developed complete occlusion of the IVC and bilateral common iliac veins, along with acute thrombosis of the femoral veins. This suggests that cases where the retrieval hook is removed but the filter does not fully open may also be considered a form of unsuccessful conversion. Once chronic IVC occlusion occurs, the obstruction becomes irreversible without stent, regardless of whether the filter can be fully expanded through subsequent surgical intervention. These patients would benefit from longer follow-up periods.

When patients are faced with chronic occlusion of IVC, as demonstrated in this case, not all are willing or suitable candidates for IVC stenting. Therefore, the most effective way to prevent such outcomes is to ensure the complete expansion of the filtering legs during conversion, ideally achieving an angle close to 0° between the filtering legs and the vein wall. Unfortunately, no current indicators predict whether the filtering legs will fully expand. Some studies have explored the relationship between fibrinogen levels and filter leg expansion after conversion, hypothesizing that fibrin may contribute to filter leg adhesion, but these studies have not found statistically significant results (11). Other factors, such as the duration of filter placement before conversion, whether the filter intercepted thrombi, the use of oral anticoagulants, and the presence of thrombophilia, have not been thoroughly studied.

These discussions focus on the impact of unexpanded filtering legs on hemodynamics. However, even when the filtering legs are fully expanded, the residual structure of the VTCF remains in the body long-term. Given that the patency rates for IVC stents used in non-thrombotic lesions are only 80% to 97%, there is reason to suspect that residual parts of the VTCF may also contribute to thrombosis formation. Currently, research on this aspect is lacking. For patients prone to thrombosis, the VTCF may not be the optimal choice.

## Conclusion

After the retrieval hook is removed, the inability of the filtering legs to fully open remains an inherent issue in the design of this filter. While in most cases, the filtering legs can be successfully opened completely, attention is still required when full expansion is not achieved. This case underscores the limited understanding of the VTCF. Both the successful removal of the retrieval hook and the complete expansion of the filtering legs appear equally critical. The management of the filtering legs should be more proactive, with every effort made during the conversion process to ensure the filter fully expands, minimizing its impact on blood flow. This is particularly important for younger patients and those with thrombophilic conditions (as in this case). If it is not possible to guarantee the filter's successful conversion and full expansion, the suitability of the VTCF for such patients should be reconsidered. Extended follow-up is essential to monitor patient outcomes and address potential issues.

## Data Availability

The original contributions presented in the study are included in the article/Supplementary Material, further inquiries can be directed to the corresponding author.
